# Bluetongue Virus Nonstructural Protein NS3/NS3a Is Not Essential for Virus Replication

**DOI:** 10.1371/journal.pone.0085788

**Published:** 2014-01-20

**Authors:** René G. P. van Gennip, Sandra G. P. van de Water, Piet A. van Rijn

**Affiliations:** Central Veterinary Institute of Wageningen UR (CVI), Department of Virology, Lelystad, The Netherlands; The Pirbright Institute, United Kingdom

## Abstract

Orbiviruses form the largest genus of the family *Reoviridae* consisting of at least 23 different virus species. One of these is the bluetongue virus (BTV) and causes severe hemorrhagic disease in ruminants, and is transmitted by bites of *Culicoides* midges. BTV is a non-enveloped virus which is released from infected cells by cell lysis and/or a unique budding process induced by nonstructural protein NS3/NS3a encoded by genome segment 10 (Seg-10). Presence of both NS3 and NS3a is highly conserved in *Culicoides* borne orbiviruses which is suggesting an essential role in virus replication. We used reverse genetics to generate BTV mutants to study the function of NS3/NS3a in virus replication. Initially, BTV with small insertions in Seg-10 showed no CPE but after several passages these BTV mutants reverted to CPE phenotype comparable to wtBTV, and NS3/NS3a expression returned by repair of the ORF. These results show that there is a strong selection for functional NS3/NS3a. To abolish NS3 and/or NS3a expression, Seg-10 with one or two mutated start codons (mutAUG1, mutAUG2 and mutAUG1+2) were used to generate BTV mutants. Surprisingly, all three BTV mutants were generated and the respective AUG^Met^→GCC^Ala^ mutations were maintained. The lack of expression of NS3, NS3a, or both proteins was confirmed by westernblot analysis and immunostaining of infected cells with NS3/NS3a Mabs. Growth of mutAUG1 and mutAUG1+2 virus in BSR cells was retarded in both insect and mammalian cells, and particularly virus release from insect cells was strongly reduced. Our findings now enable research on the role of RNA sequences of Seg-10 independent of known gene products, and on the function of NS3/NS3a proteins in both types of cells as well as in the host and insect vector.

## Introduction

Orbiviruses form the largest genus of the family *Reoviridae* consisting of at least 23 virus species [1]. Three of these orbivirus species, bluetongue virus (BTV), epizootic haemorrhagic disease virus (EHDV), and African horsesickness virus (AHSV) cause a ‘notifiable disease’ as listed by the Office International des Epizooties (OIE) [2]. Virus transmission between ruminants (BTV and EHDV) or equids (AHSV) occurs in majority by bites of specific species of *Culicoides*. BTV is causing severe haemorrhagic disease in ruminants with fever, lameness, coronitis, swelling of the head (particularly the lips and tongue) and death. BTV has been the most extensively studied orbivirus and serves as representative of arthropod-borne orbiviruses. Twenty-four serotypes of BTV have been recognized as defined by cross-neutralization assays, and recent BTV isolates are considered as serotype 25 and 26 which was partially based on sequence data [3], [4].

The genome of orbiviruses consist of ten linear double-stranded RNA genome segments (Seg-1 to Seg-10) encoding structural proteins VP1 to VP7, non-structural proteins, NS1, NS2 and NS3/NS3a, for reviews see [5], [6], and the recently discovered non-structural protein NS4 of BTV [7], [8]. The virus particle composes three shells of proteins. The inner shell consists of VP3 encoded by Seg-3, the middle shell consists of VP7 encoded by Seg-7, and the outer shell is formed by VP2 (Seg-2) and VP5 (Seg-6). The orbivirus particle further contains enzymatic proteins VP1 (Seg-1), VP4 (Seg-4) and VP6 (Seg-9), and one copy of each of the ten genome segments in the inner shell. The non-structural proteins NS1 (Seg-5), NS2 (Seg-8), NS3/NS3a (Seg-10), and NS4 (Seg-9) are not part of the virus particle.

**Figure 1:**
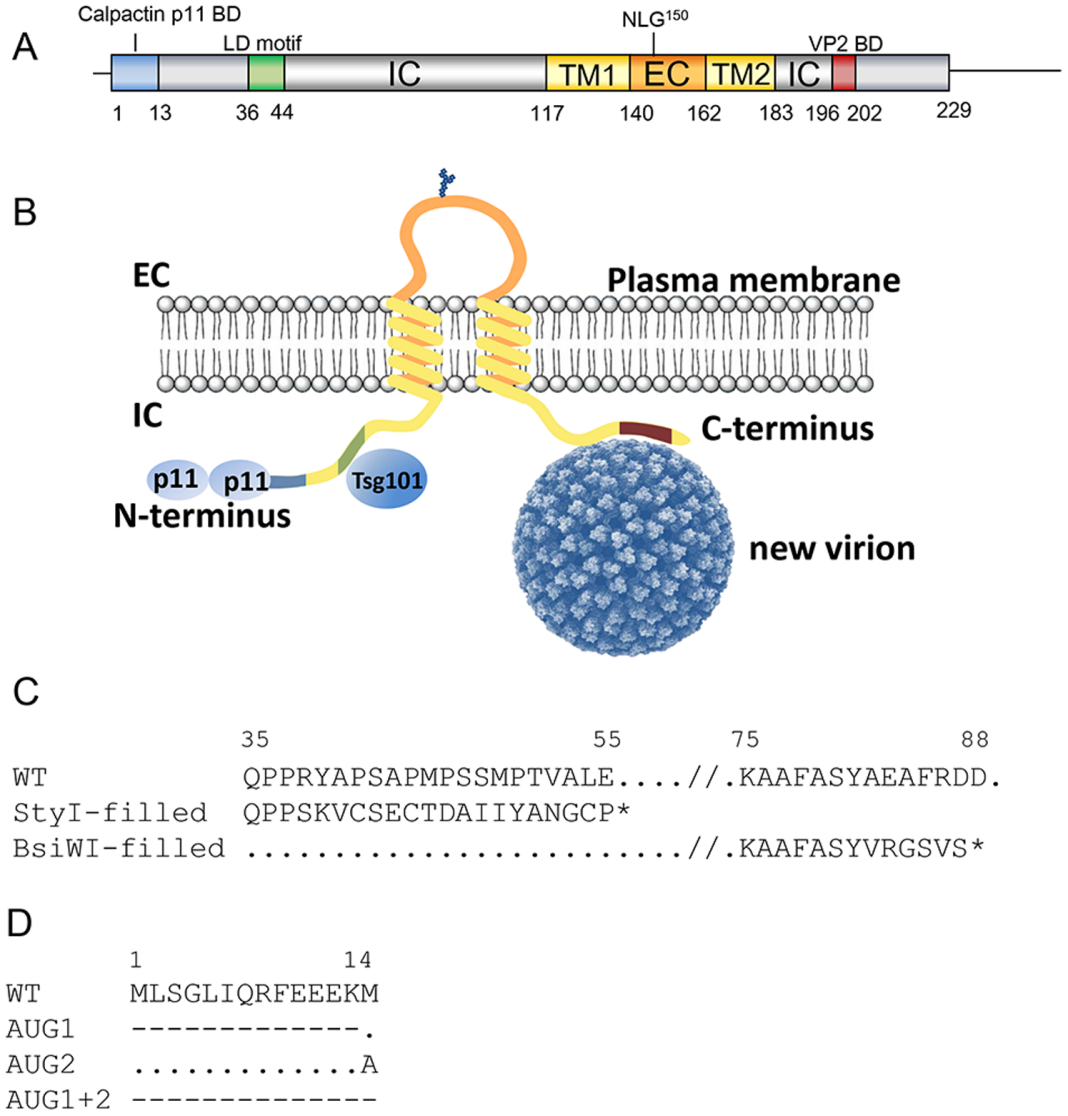
Schematic representation of BTV NS3 and putative amino acid sequences of mutant viruses. (A) Schematic representation of BTV NS3. Numbers indicate amino acid position according to the NS3 amino acid sequence The two cytoplasmic domains (IC), the two transmembrane domains (TM; yellow), the extracellular domain (EC; orange), the calpactin S100A10/p11 binding site (blue), the late domain motifs (LD; green), the VP2 binding domain (VP2 BD: red), and the conserved N-linked glycosylation site at position 150 (NLG) are indicated. (B) NS3 is an integral membrane protein that interacts to cellular release factors calpactin S100A10/p11 and Tsg101, whereas the C-terminal domain binds VP2 on the outside of the virus particle. (C) The putative amino acid sequence in the regions of the *Sty*I, *Bsi*WI sites and StyI-filled or BsiWI-filled are shown in single letter code. Changed amino acids by a 4-basepairs insertion are underlined. (D) Single and double AUG→GCC mutations are shown. Dots and dashes indicate no change and absence of expression, respectively.

Cell lysis is considered as the predominant mechanism in the release of non-enveloped viruses into the environment. As for other non-enveloped viruses like SV40 [9] and poliovirus [10], complex trafficking and budding strategies are also involved in release of orbiviruses which is likely mediated by the NS3/NS3a protein. BTV releases the cell by both mechanisms of which cell lysis is the major mechanism in mammalian cells, whereas no cell lysis after infection of insect cells has been observed [11], [12], [13]. Virus release occurs by budding of the cell membrane therewith acquiring a temporary envelope or by disruption of the cell membrane. Non-lytic release of progeny virus from infected cells has been supposed to be mediated by NS3/NS3a encoded by Seg-10 [14]. In contrast to expression in mammalian cells, NS3/NS3a is highly expressed in *Culicoides* cells. Remarkably, NS3/NS3a of the non-enveloped orbiviruses are membrane associated glycosylated proteins [15]. The proteins contain two transmembrane regions flanked by a long N-terminal and short C-terminal cytoplasmic domain, and a small extracellular domain with a highly conserved N-glycosylation site between both membrane regions (Fig. 1). Further, the N-terminal part of NS3, not present in NS3a, interacts with cellular release factors calpactin S100A10/p11 and Tsg101, whereas the C-terminal domain binds VP2 on the outside of the virus particle [16], [17], [18]. The amino acid sequence of NS3/NS3a varies however considerably between and within different orbivirus species, but the abovementioned motifs and domains are well conserved. The function of NS3a of orbiviruses remains unclear, although the second in-frame start codon of NS3a is completely conserved in the major arthropod-borne orbivirus species, suggesting an important role for NS3a in the mammalian or insect cell.

NS3/NS3a protein exhibits viroporin-like properties, and these proteins have been extensively studied to elucidate their role in virus replication [19]. NS3/NS3a complementing cell lines have been used to produce NS3/NS3a mutants of BTV suggesting an essential role in BTV replication in mammalian cells [17]. Recently, reverse genetics for cell-adapted BTV1, vaccine virus for serotype 6 as well as for virulent BTV8 has been developed [20], [21]. Here, we have used reverse genetics to generate NS3/NS3a mutant viruses to investigate the role of NS3/NS3a in BTV replication. Both NS3 and NS3a seemed to be involved in virus release from insect cells, whereas this effect was less obvious for NS3a in mammalian cells. More importantly, although considered to be essential, expression of NS3 and NS3a is not required for virus propagation in both mammalian and insect cells.

## Materials and Methods

### Cell Lines, Viruses, and Antibodies

BSR cells (a clone of BHK-21 cells [22]) were cultured in Dulbecco’s modified Eagle’s medium (DMEM; Invitrogen) containing 5% fetal bovine serum (FBS), 100 IU/ml penicillin, 100 µg/ml streptomycin and 2,5 ug/ml Amphotericin B.

*C. variipennis* (KC) cells [13] were grown in modified Schneider’s Drosophila medium with 15% heat inactivated foetal bovine serum, 100 IU/ml penicillin and 100 µg/ml streptomycin.

All viruses used in this study were generated by reverse genetics. Virus stocks were obtained by infection of BSR cells at low multiplicity of infection (MOI) and harvested when 100% cytopathogenic effect (CPE) was observed. Virus titers were determined by endpoint dilution and were expressed as 50% tissue culture infective dose (^10^logTCID_50_/ml). Virus stocks were stored at −80°C.

Monoclonal antibody (Mab) directed against BTV-VP7 was produced by hybridoma ATCC-CRL-1875 (American Type Culture Collection). Mabs 32b6, 32h2 and 32f1 directed against NS3 were a gift from Ingenasa, Spain. Polyclonal antibodies (Pabs) against VP5 (gift from Michiel Harmsen CVI-Lelystad) were raised against peptide 208–245 of BTV8-VP5 (Genscript corporation; Piscataway, NJ).

### Construction of Mutant Genes of Seg-10

Mutations in Seg-10 of BTV8 [21] were introduced by filling in the recognition sites for *Sty*I (at nucleotide position 129) or *Bsi*WI (at nucleotide position 259) with Klenow large fragment of DNA polymerase followed by ligation resulting in a 4-basepairs insertion. Start codons 1 and 2 were mutated (AUG→GCC) in Seg-10 of BTV8. Therefore, cDNAs of fragments containing the AUG→GCC mutation (mutAUG1 or AUG2) were synthesized by Genscript corporation (Piscataway, NJ) and cloned in pUC57. These fragments were exchanged in pUC57 containing Seg-10 of BTV8 by standard methods resulting in mutAUG1 and mutAUG2 under control of the T7 RNA-polymerase promoter and a recognition site for a restriction enzyme at the 3′-terminus for defined run-off transcription. Start codon mutations were combined by exchange of the *Bst*BI fragment of mutAUG2 in the plasmid containing mutAUG1 resulting in mutAUG1+2.

All cDNAs were completely sequenced to confirm the sequence and respective start codon mutations. Plasmids were maintained in *E.coli* DH5α, and were purified using the QIAfilter Plasmid Midi Kit (Qiagen).

### Rescue of BTV from RNA Transcripts

T7-derived RNA transcripts from linearized plasmids were synthetized and generating of BTV was performed as previously described [21]. Briefly, monolayers of 10^5^ BSR cells per 2 cm^2^ were transfected with equimolar amounts of RNA of BTV segments encoding VP1, VP3, VP4, NS1, VP6, NS2. In total, 600 ng RNA was transfected using 1,5 µl lipofectamine™ 2000 (1∶2.5; 1 mg/ml Invitrogen) in Opti-MEM® I Reduced Serum Medium according to manufacturer’s conditions. Eighteen to twenty hours post transfection, monolayers were transfected again with 600 ng equimolar amounts of ten BTV RNA segments. BTV mutants were rescued from T7-derived RNA transcripts using Seg-1 to 9 from BTV1 completed with Seg-10 (mutated) RNA from BTV8. Supernatants were harvested from monolayers 48 h after the second transfection. Cytopathogenic effect (CPE) specific for BTV was confirmed by immunostaining of fixed monolayers with VP7 directed Mab CRL-1875 according to standard procedures. If no clear CPE was observed, but immunostaining was positive, duplicate wells were passed in 1∶5 dilution and screened for BTV replication by CPE and/or immunostaining.

### Sequencing of Seg-10

Viral RNA was isolated from 200 µl of virus stocks with the High Pure Viral RNA kit (Roche). Seg-10 was amplified with primers NS3-S10f* (5′-GTTAAAAAGTGTCGCTGCC-3′) and NS3-S10r (5′-GTAAGTGTGTAGTGTCGCGCAC-3′). Six µl of RNA was denaturated at 94°C for 3 min and immediately cooled on ice. A one-step RT-PCR kit (Qiagen) was used to reversely transcribe RNA and subsequently cDNA was amplified in a RT-PCR containing both primers. The reaction mix contained 10 µl of 5×QIAGEN one-step RT-PCR buffer, 2 µl of dNTP mix, 0.6 µM of each primer and 2 µl of the enzyme mix (containing RT and PCR reaction enzymes). RNase-free water was added to a total volume of 44 µl. Six microliters of denatured RNA was added to the mix. The RNA was reversely transcribed at 45°C for 30 min. This was followed by an activation step at 94°C for 15 min. Forty amplification cycles were then carried out (94°C for 1 min, 45°C for 1 min and 72°C for 2 min, followed by a terminal extension step at 72°C for 10 min. The cDNA products were analyzed by 0.9% agarose gel electrophoresis and visualized under UV light after staining with ethidium bromide.

Amplicons were completely sequenced to confirm Seg-10 sequences using appropriate primers and the BigDye® Terminator v1.1 Cycle Sequencing Kit in a ABI PRISM® 3130 Genetic Analyzer (both supplied by Applied Biosystems, Foster City, IA, USA).

### Growth Curve on BSR and KC Cells

Confluent BSR- or KC-monolayers in M24-well plates were infected in duplicate at a multiplicity of infection (MOI) of 0.01. After attachment to cells for 1.5 h at 37°C, the medium was removed (and washed once) and refreshed with 1 ml of DMEM complete medium (see cell lines) and incubation was continued. At 6, 16, 24, 30, 40, 72 and 96 h post infection (hpi), supernatants and cells were separated, harvested and stored at −80°C. Virus titers were determined by endpoint dilution on BSR cells expressed as tissue culture infective doses (^10^logTCID_50_/ml). Therefore, BSR cells were infected with tenfold dilutions of samples, and grown for 72 h. Positive wells were detected by immunostaining with VP7-specific Mab ATCC-CRL-1875.

### Plaque Morphology of BTV on BSR Cells

BSR monolayers were infected by tenfold dilutions of indicated viruses, and grown for 48 h under overlay medium (K1000 complete with 1% methylcellulose). Monolayers were fixed with methanol/acetone (1∶1) and immunostained with Mab ATCC-CRL-1875. Plaques in appropriate dilutions were compared.

### Western Blot Analysis of NS3/NS3a Proteins

BSR cells were infected with indicated viruses with an MOI of 0.1. Lysates were prepared from 25 cm^2^ flasks with beginning CPE throughout the whole monolayer. Therefore, cells were lysed in 2 ml PBS containing 1% NP40 and protease inhibitor cocktail complete (Roche, 1 tablet/10 ml lysis buffer). Cell debris was removed by centrifugation 5′ 4000 rpm at 4°C. For western blotting, lysates were concentrated using Amicon Ultracel® 10K spin columns (Millipore). Ten µl samples in LDS buffer (NuPAGE) with reducing agent (NuPAGE) were heated for 2′ at 70°C and separated by electrophoresis on a 12% BIS-TRIS polyacrylamide-gel in 1× MOPS-SDS buffer using the XCell-Surelock system. Separated proteins were transferred to nitrocellulose paper. Transferred proteins were incubated with Mabs/Pabs and as second antibody horse-radish peroxidase (HRPO)-conjugated rabbit α-mouse (for Mabs against BTV-NS3), goat α-rabbit (for Pabs against BTV-VP5) and rabbit α-goat (for goat serums against BTV8) were used according to manufacturer’s conditions. Bound conjugates were detected with Supersignal (Pierce) by exposure to medical X-ray films (Fuji).

## Results

### Repair of NS3/NS3a Expression in BTV Mutants

**Figure 2:**
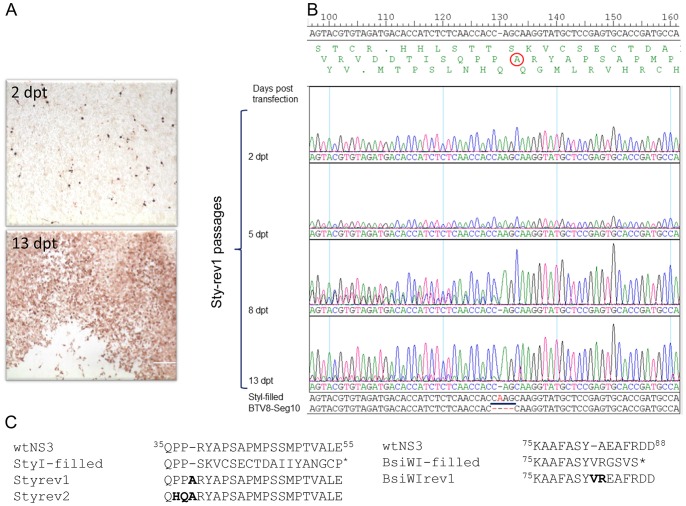
Analysis of revertant viruses Sty-rev and BsiWI-rev. (A) anti-VP7 immunostained cells after 2 or 13 days post transfection with BTV1 Seg-1-9 and mutated Seg-10 StyI-filled resulting in revertant virus StyI-rev1 showing CPE at 13 dpt. (B) sequence analysis of Seg-10 amplicons from StyI-rev1 passages at different time points after transfection. In single letter code, putative translation of all three frames is shown of which the middle is the ORF of NS3 with the insertion of Alanine (A) in StyI-rev1. Here the analysis is shown by use of a sequence primer located downstream of the *Sty*I site. Note the mixed sequence at 8 dpt upstream of the filled site due the introduction of the point deletion at the *Sty*I site in a subpopulation of the fragments. (C) Comparison of the amino acid sequences of NS3 of wtBTV1/8(S10), the 4-basepairs insertions, and the rescued revertant viruses Sty-rev1, 2 and BsiWI-rev1. Amino acids changes in the regions of the 4-basepairs insertion of the revertant viruses Sty-rev1, 2 and BsiWI-rev1 are in bold.

The role of different domains of NS3/NS3a (Fig. 1a) in trafficking and egress of BTV was studied by 4-basepairs insertions at different positions in Seg-10. Filling in of the *Sty*I site resulted in truncated NS3/NS3a at amino acid position 56 (Fig. 1C) located in the first of the two late domain motifs. Similarly, a 4-basepairs insertion was introduced at the *Bsi*WI site resulting in truncated NS3/NS3a at position 88 (Fig. 1C) between the late domain and the first transmembrane region. Using these mutated segments in reverse genetics to generate BTV mutants no CPE was detected two days after the second RNA transfection (2 dpt). However, cells were immunostained with VP7 Mab indicating expression of viral proteins (Fig. 2A; 2 dpt). Transfected cells of duplicate wells were passaged and at 8 dpt distinct plaques of immunostained cells were visible for both Seg-10 mutants suggesting BTV replication. After a subsequent passage, CPE was shown for the *Sty*-filled mutation in Seg-10 (Fig. 2A; 13 dpt). Sequencing of Seg-10 of this passage showed a point deletion resulting in repair of the open reading frame (ORF) of NS3/NS3a thereby inserting an Alanine at position 38 of NS3 (StyI-rev1, Fig. 2B). Reverse genetics with this mutated Seg-10 was repeated and a BTV revertant was generated again after the same number of passages (StyI-rev2). Sequence results showed again that the ORF of NS3/NS3a was restored by a point deletion. The putative amino acid sequence of mutated NS3/NS3a of StyI-rev2 compared to wild type NS3/NS3a showed, in addition to an extra alanine, a double amino acid mutation (PP→HQ) (Fig. 2C). Noteworthy, the conserved first late domain motif PPRY was mutated in both StyI-revertants.

Duplicate wells of the *BsiW*I mutated Seg-10 were also passaged, since cells were consistently immunostained suggesting BTV replication. The BsiWI-filled mutant reverted from non-CPE to CPE phenotype in nine subsequent passages. Sequencing of Seg-10 revealed a point deletion, resulting in restoration of the ORF of NS3/NS3a by insertion of valine at position 82 and one amino acid change (A→R) at position 83 (Fig. 2C). Apparently, BTV replication strongly favours expression of NS3/NS3a, since 4-basepairs insertions in the ORF were restored and virus was rescued which was associated with a phenotypical change from non-CPE to CPE despite of several amino acid mutations.

### NS3 and NS3a are not Essential for BTV Replication

In order to study the role of NS3 and NS3a in BTV replication, expression of NS3 and NS3a from Seg-10 was separated by single AUG→GCC mutations in Seg-10 (mutAUG1 and mutAUG2, respectively). In addition, both start codon mutations were mutated to study the role of both proteins (mutAUG1+2). MutAUG1 would not lead to expression of NS3 but only NS3a and mutAUG2 would only express NS3and not NS3a, whereas mutAUG1+2 will not translate both NS3 and NS3a protein (Fig. 1D). All three mutated Seg-10′s were used to generate BTV mutants using reverse genetics. At 2 dpt clear CPE was observed for mutAUG2, whereas delayed CPE was observed after further passaging of transfected cells (p2) for mutAUG1. Similarly, mutAUG1+2 virus was detected in supernatant of transfected cells at 8 dpt after two passages of transfected cells. Delayed CPE similar to mutAUG1 was observed in BSR cells for mutAUG1+2 after infection of fresh monolayers. Surprisingly, all three BTV mutants were generated from which we conclude that neither NS3 nor NS3a is essential for BTV propagation.

Extensive studies were performed to confirm the absence of both proteins in propagated BTV mutants.

**Figure 3:**
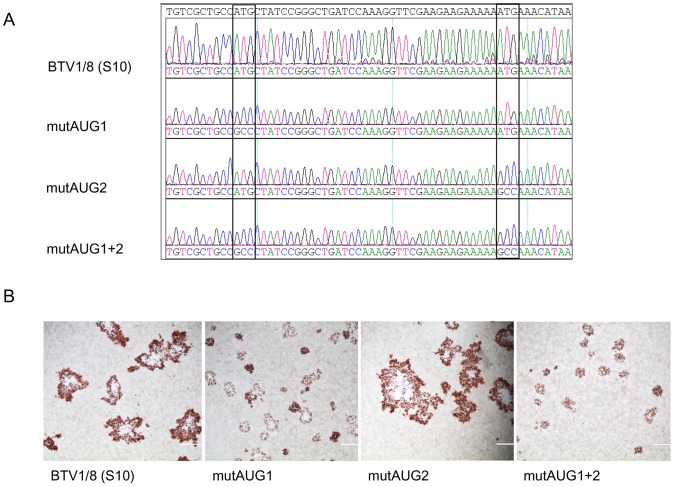
Sequence analysis and plaque phenotype of AUG mutant viruses. (A) Sequence analysis of Seg-10 amplicons from AUG mutant viruses shown in DNASTAR Lasergene Seqman assembly software. The mutated codons are indicated in rectangles (B) Plaque morphology of BSR cells infected with wtBTV1/8(S10), mutAUG1, mutAUG2 and mutAUG1+2 virus grown under 1% methylcellulose overlay medium are shown. At 2 dpi cells were fixed and immunostained with anti-VP7 Mab.

Sequence analysis of entire Seg-10 RNA isolated from supernatants containing infectious BTV confirmed the presence of the respective AUG→GCC mutations in all three BTV mutants. (Fig. 3A). BTV mutants were reproducibly generated, and only one out of three mutAUG1 virus stocks showed an additional point mutation at position 486 in Seg-10 resulting in a V→A mutation at aa position 156 of NS3. This amino acid mutation did not result in a difference in plaque size (data not shown). All other independently generated AUG mutant viruses did not contain additional mutations in Seg-10.

**Figure 4:**
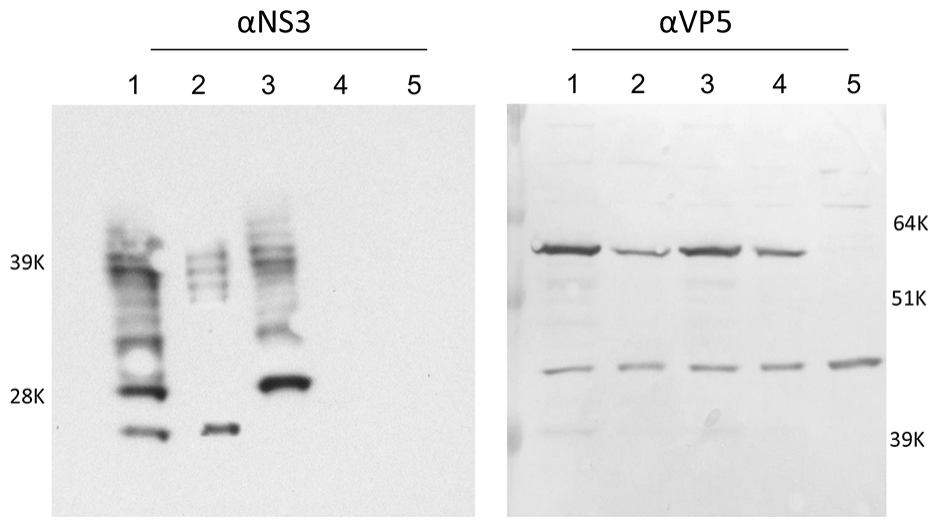
Detection of viral proteins of AUG mutant viruses. Westernblot analysis of expression of NS3 and VP5 in cell lysates from BSR cells infected with wtBTV1/8(S10) (lane 1), mutAUG1 (lane 2), mutAUG2 (lane 3), mutAUG1+2 (lane 4) and mock-infected cells (lane 5) using antibodies against BTV NS3 or VP5.

Expression of NS3 and/or NS3a in infected cells was detected by immunostaining with NS3/NS3a specific Mabs for both single start codon mutations, but not for mutAUG1+2 virus. Infection of mutAUG1+2 virus was however confirmed by immunostaining with anti-VP7 Mab (Fig. 3B). NS3/NS3a expression was further investigated in concentrated lysates by western blotting using anti-NS3/NS3a Mabs. Cells infected with mutAUG1 virus expressed a protein comigrating with the smaller band (NS3a) of the two NS3-related proteins expressed in BSR cells infected with wtBTV1/8 (S10) (Fig. 4, left panel lanes 1 and 2). Cells infected with mutAUG2 virus only expressed NS3 comigrating with the larger NS3-related protein (Fig. 4, left panel lanes 1 and 3). No NS3-related proteins were detected in cells infected with mutAUG1+2 virus indicating that truncated forms of NS3 were not expressed (Fig. 4, left panel, lane 4). Further, lanes 1–3 with NS3-related proteins showed protein bands of higher molecular weight representing different forms of the carbohydrate groups of NS3/NS3a (lane 1–3). The protein bands were not present in lane 4 (mutAUG1+2) confirming that this BTV mutant is not expressing NS3/NS3a or any other truncated form of NS3. VP5 protein was detected in all (wtBTV1/8(S10), mutAUG1, mutAUG2 and mutAUG1+2) infected cells demonstrating expression and detection of virus proteins, and confirmed infection by the respective BTV mutants (Fig. 4, right panel, lanes 1–4).

### NS3 and NS3a are Involved in Cytopathogenic Effect and Virus Release

A clear difference in CPE was observed between wtBTV1/8(S10) and mutAUG2 compared to mutAUG1 and mutAUG1+2 (Fig. 3b). These two groups of two viruses also differ in expression of NS3; wtBTV1/8(S10) and mutAUG2 are expressing NS3, whereas mutAUG1 and mutAUG1+2 are not expressing NS3. Apparently, the CPE phenotype is correlated to NS3 expression, whereas expression of NS3a (mutAUG1 versus mutAUG1+2) showed no obvious difference in CPE in BSR cells.

**Figure 5:**
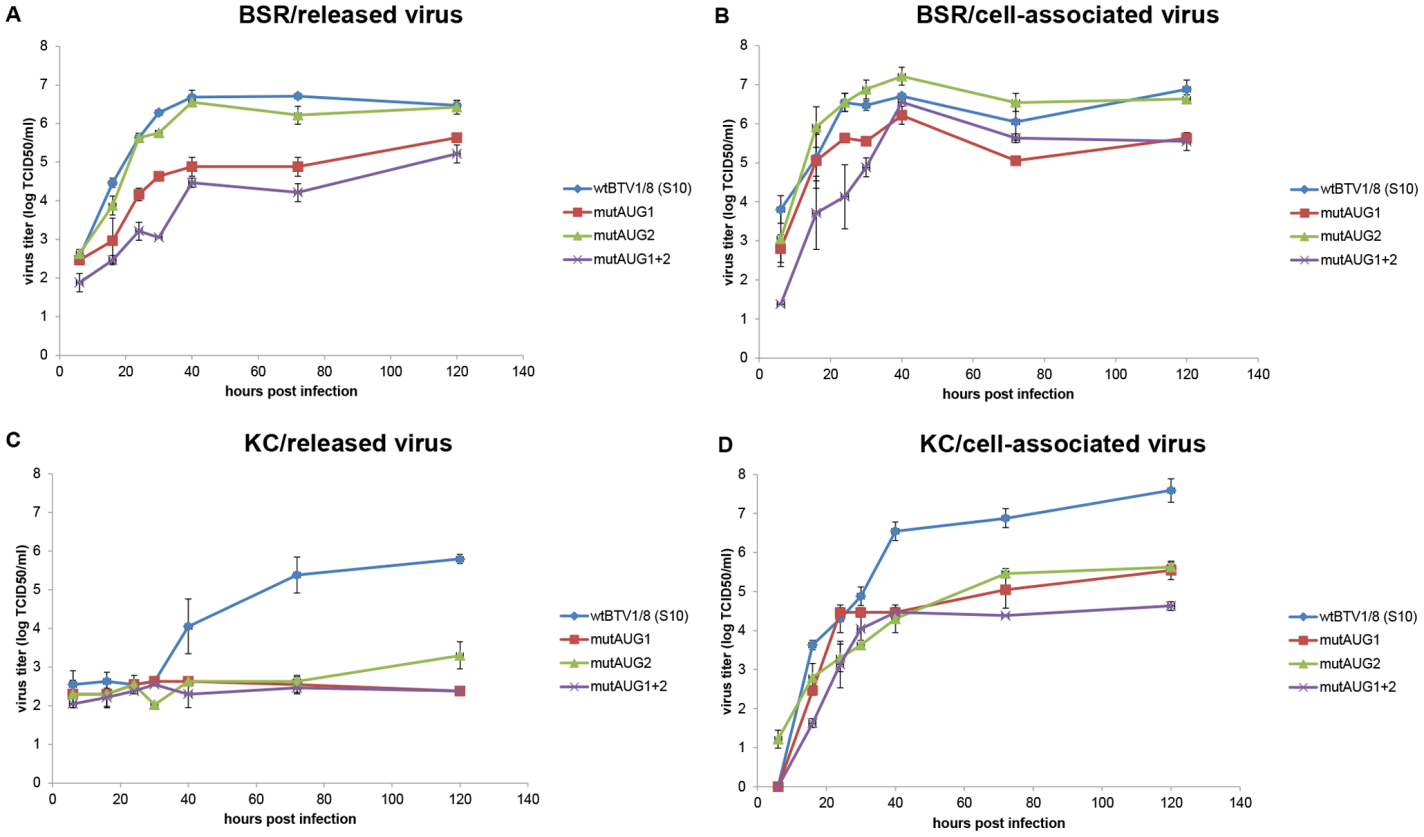
Replication kinetics of AUG mutant viruses on mammalian and insect cells. Virus growth and virus release of AUG mutant viruses grown on BSR cells and KC cells infected in duplicate with wtBTV1/8 (S10), mutAUG1, mutAUG2 and mutAUG1+2 viruses and harvested at indicated time points post infection. Virus titers were determined in supernatant and in cells by endpoint dilution, and the mean values are expressed as ^10^logTCID_50_/ml. (A) released virus from BSR cells (upper left panel) or (B) BSR cell-associated virus (upper right panel). (C) released virus from KC cells (lower left panel) or (D) KC cell-associated virus (lower right panel).

Since BTV mutants lacking NS3 expression showed a smaller plaque size, this could affect virus growth or release of virus into the culture medium. Therefore growth of these virus mutants was studied in BSR and KC cells (Fig. 5a–d). Monolayers were infected with an MOI of 0.01 and virus titers were determined in supernatant (released virus) and cells (cell-associated virus) at different time points after infection. All viruses reached maximum virus titers in mammalian cells (BSR) of approximately 6–7 ^10^logTCID_50_/ml at 40 hours post infection (hpi) (Fig. 5a/b). Maximum virus titers in supernatant for wtBTV1/8(S10) and mutAUG2 virus coincided with these in the cells, whereas virus titers for mutAUG1 and mutAUG1+2 levels were still increasing after 120 hpi. These results indicate that BTV mutants without NS3 are hampered in release of virus from mammalian cells.

Growth curves in insect cells (KC) showed cell-associated virus titers increasing rapidly in 20–30 hpi and these virus titers reached a maximum of approximately 7.8 ^10^logTCID_50_/ml for wtBTV1/8(S10), 5.7 for mutAUG1 and mutAUG2, and 4.6 (mutAUG1+2) (Fig. 5c/d). wtBTV1/8(S10) was released into the supernatant from KC cells at 40 hpi to a virus titer of 4.55 ^10^logTCID_50_/ml and further increased slowly to maximum virus titers of 5.9 ^10^logTCID_50_/ml at 120 hpi. MutAUG2 (also expressing NS3) showed a strongly delayed virus release of ^10^log 3.6 TCID50/ml in supernatant at 120 hpi. In contrast, both mutAUG1 and mutAUG1+2 (not expressing NS3) were not significantly secreted into the medium. For these mutants, the amount of cell-associated virus was comparable in the first 24 hours and then continued at a maximum in the following harvests up to 120 hpi. Apparently, virus release from insect cells (KC) of BTV mutants without NS3 expression was strongly reduced. In mutAUG2, only NS3 is expressed and showed a very strong delay in virus release at 120 hpi compared to wtBTV1/8(S10) expressing both NS3 and NS3a. These results suggest a role of NS3a in virus release from insect cells.

## Discussion

The mechanisms of virus release from the infected cell of arthropod borne viruses is very intriguing, since it might be linked to pathogenesis and viremia as well as to transmission of virus between host and insect vector. Nonstructural proteins NS3 and NS3a of BTV encoded by Seg-10 are involved in trafficking and egress of virus [17], [18]. The calpactin light chain (S100A10/p11) binding domain on the N-terminal end of NS3 is involved in intracellular trafficking of BTV [18], and NS3/NS3a also interacts with Tsg101 and uses the ESCRT pathway in mammalian cells for budding [23]. Further, the C-terminal domain interacts with VP2 on the outside of the BTV particle [16].

Here, we used the established reverse genetics system for BTV [21], [24] to investigate the role of motifs and domains of NS3/NS3a in BTV replication. Intending to express C-terminally truncated NS3/NS3a proteins, 4-basepairs insertions were introduced at different positions in the ORF of NS3/NS3a resulting in out-of-frame mutations. However, generated BTV mutants showed a point deletion therewith restoring the expression of NS3/NS3a with an insertion of one codon at the position of the original 4-basepairs insertion. For another revertant, the point deletion also resulted in amino acid mutations upstream the inserted codon (Fig. 2). Obviously, mutations restored the expression of NS3/NS3a and this expression is associated with CPE. Thus, despite of amino acid mutations in NS3/NS3a, including mutations in the first late domain, expression of NS3/NS3a is highly favorable in BTV propagation. Apparently, BTV revertants expressing NS3/NS3a were selected by passing the transfected cells and this procedure generated faster replicating BTV mutants that overgrow the original BTV mutant (Fig. 2). This method can be used to study the role of other regions and domains in NS3/NS3a, and herewith the mechanism of mutagenesis by the RNA dependent RNA polymerase of orbivirus.

Revertants of NS3/NS3a mutations suggested a very important or even essential role of NS3/NS3a. This is in agreement with an earlier report in which a similar NS3 mutant (named BTVM1 in that study) was generated using *in trans* complementation of NS3/NS3a proteins [18]. Nonetheless, we were able to generate BTV mutants lacking expression of NS3, NS3a or both, and propagate these BTV mutants in normal BSR cells (mutAUG1, mutAUG2, and mutAUG1+2). Moreover, these BTV mutants were analyzed by several methods to confirm expression of the respective NS3 and NS3a proteins. Besides initiation codons AUG1 and AUG2 for expression of NS3 and NS3a respectively, another eleven in-frame AUG codons are present in Seg-10. So, it cannot be excluded that scanning ribosomal subunits could use downstream located AUG codons as initiation codon resulting in N-terminally truncated NS3 proteins. We could not detect any NS3 related proteins from the mutAUG1+2 by immunostaining of fixed cells and Westernblot analysis indicating the absence of NS3, NS3a or other NS3 related proteins, provided that binding of antibodies to these products is not disturbed. We conclude that mutAUG1+2 virus represents BTV with a nontranslated genome segment 10 with respect to NS3/NS3a expression, and that neither NS3 nor NS3a is essential for virus propagation. Although expression of other yet unknown gene products by Seg-10 is most unlikely the existence cannot be ruled out.

Still, the presence of NS3 and NS3a proteins encoded by Seg-10 is strongly conserved in *Culicoides* arthropod borne orbiviruses supporting an important role for both of these proteins. The amino acid sequence between the NS3 and NS3a initiation codons of orbiviruses is variable in length, but is highly conserved in length and sequence within studied orbiviruses species like BTV and AHSV. This region, harboring the calpactin light chain (S100A10/p11) binding domain essential for intracellular trafficking of BTV in BSR cells, is lacking in NS3a [18]. Obviously, the main mechanism of egress of non-enveloped viruses is cell lysis, therewith most likely killing the infected mammalian cell. However, non-lytic processes of virus release, like budding, have also been suggested [25], [26]. For arthropod borne orbiviruses, cell lysis has not been observed in insect cells in contrast to cell lysis of mammalian cells [11], [12], [13], [27]. BTV release from BSR cells is mainly dependent on CPE through the permeabilization of the membrane likely induced by NS3 [19], whereas BTV release from KC cells is dependent on budding [17]. Virus release from BSR cells is solely dependent on NS3 expression, whereas virus release from KC cells is strongly reduced by either lack of NS3, NS3a or both proteins (Figure 5). Since virus release from insect cells occurs by budding, these results suggest that both proteins are involved in budding, and thus also suggests a specific role for NS3a. This might also explain the conserved second start codon in Seg-10 of arthropod borne orbivirus species. Further research is needed to unravel the specific role of each of these non-structural proteins in virus release from insect cells.

Generally, replication of all BTV mutants was similar to wtBTV1/8(S10) in KC and BSR cells for the first 24 hours post infection but after this period, differences were observed in released virus as well as in cell-associated fractions. Inefficient release of virus appeared to coincide with lower virus titers in cells in both cell types. Thus, the total virus production is lower for mutants disturbed in virus release. Apparently, accumulation of virus in the cytosol by lack of transport to the membrane and subsequent egress of virus will decrease or delay re-infections after the first round of infection. However, this accumulation could also hinder BTV replication in the infected cell. For BSR cells, a lower virus titer in the supernatant and cell-associated fraction was also associated with a significantly delayed but clear CPE and reduced plaque size. Celma *et al.* [18] did not observe CPE of BSR cells infected with BTVM14, which is similar to our mutAUG1 virus, and these authors have suggested a blockade in BTV replication late in infection, since all other viral processes were accomplished like protein translation, genome replication and assembly of viral cores [18]. We indeed suggest that disturbing the virus release also reduce BTV replication in BSR cells, and causes delayed CPE and reduced plaque size. However, this disturbed virus release is not a complete blockade, since mutant viruses were propagated, and successfully passed in normal cell lines.

Obviously, NS3 plays an important role in BTV release from mammalian cells, whereas both NS3 and NS3a are important for release from KC cells. However, both NS3 and NS3a can be deleted and resulted in replicating BTV without NS3/NS3a expression. In conclusion, mutAUG1+2 virus can still cause CPE, and we assume that CPE is the main mechanism of egress to the medium of BTV lacking NS3/NS3a protein. NS3 represents viroporin-like properties [19]. Viroporins compose a group of small hydrophobic transmembrane proteins that can form hydrophilic pores through lipid bilayers. Viroporins have been implicated in promoting virus release and in affecting cellular functions including protein trafficking and membrane permeability thereby promoting viral pathogenicity. Compounds interfering with the permeabilizing ability of viroporins inhibit virus production [28], [29]. This is in agreement with our results and suggests that NS3/NS3a of orbiviruses is a virulence marker as shown [30]. Moreover, viroporin-defective viruses are being explored as live-attenuated vaccines [31].

The finding of BTV propagation without NS3 or NS3a enables further research using reverse genetics on translation and functions of NS3 and NS3a in mammalian and insect cells, and in the host and the insect vector. Further, BTV with a nontranslated segment - since NS3 and NS3a are the only known gene products encoded by Seg-10 -, enables research to study the role of RNA sequences in BTV replication without interference of gene products.
